# Relationship between lifestyle proxies of cognitive reserve and cortical regions in older adults

**DOI:** 10.3389/fpsyg.2023.1308434

**Published:** 2024-01-05

**Authors:** Kristine Šneidere, Nauris Zdanovskis, Sara Mondini, Ainars Stepens

**Affiliations:** ^1^Department of Health Psychology and Pedagogy, Riga Stradiņš University, Riga, Latvia; ^2^Military Medicine Research and Study Centre, Riga Stradiņš University, Riga, Latvia; ^3^Department of Radiology, Riga Stradiņš University, Riga, Latvia; ^4^Department of Radiology, Riga East University Hospital, Riga, Latvia; ^5^Department of Philosophy, Sociology, Education and Applied Psychology, University of Padova, Padova, Italy

**Keywords:** cognitive reserve, lifestyle, occupational complexity, cortical volume, segregation approach, magnetic resonance imaging

## Abstract

**Introduction:**

With the rapid increase in the population over 65 years old, research on healthy aging has become one of the priorities in the research community, looking for a cost-effective method to prevent or delay symptoms of mild cognitive disorder or dementia. Studies indicate that cognitive reserve theory could be beneficial in this regard. The aim of this study was to investigate the potential relationship between lifestyle socio-behavioral proxies of cognitive reserve and cortical regions in adults with no subjective cognitive decline.

**Methods:**

Overall, 58 participants, aged 65–85 years, were included in the data analysis (*M *= 71.83, SD = 5.02, 20.7% male). Cognitive reserve proxies were measured using the Cognitive Reserve Index questionnaire, while cortical volumes were obtained with the Siemens 1.5 T Avanto MRI scanner and further mapped using the Desikan-Killiany-Tourville (DKT) Atlas. Estimated intracranial volume and age were used as covariates.

**Results:**

The results indicated that higher occupational complexity was associated with larger cortical volume in the left middle temporal gyrus, the left and right inferior temporal gyrus, and the left inferior parietal lobule, while a combined proxy (the total CRI score) showed a positive relationship with the volume of left middle temporal gyrus and inferior parietal lobule, and pars orbitalis in the right hemisphere.

**Discussion:**

These results might indicate that more complex occupational activities and overall more intellectually and socially active life-style could contribute to better brain health, especially in regions known to be more vulnerable to Alzheimer’s disease.

## 1 Introduction

With the life expectancy showing a trend toward increasing in European Union, there is also a trend toward a higher prevalence of dementia (Eurostat, 2022). The World Health Organization (Ageing and Health, n.d.) predicts that the number of people aged 60 or older will increase up to 2.1 billion by the year 2050. Although a longer life span is associated with a decline in different domains of daily life, evidence suggests that the actual cognitive decline and potential neurodegeneration are not associated simply with chronological age and the expression of symptoms could differ due to genetic, environmental, lifestyle and other factors (Dziechciaż and Filip, 2014; Livingston et al., 2020), thus different measures should be taken to lessen the predicted burden of pathological aging.

Cognitive and brain aging often involves both—psychological and neurobiological changes that are present in both—normal or healthy aging and pathological aging. Generally, even with normal aging thinning of the cortex, degradation of white matter and gyrus, widening of the ventricles (Blinkouskaya et al., 2021), degradation of hippocampus (Bettio et al., 2017) and other changes can be detected. These changes subsequently can lead to decline in executive function (Ferguson et al., 2021), episodic memory (Lalla et al., 2022), working memory (Pliatsikas et al., 2019) and other domains. Meanwhile, pathological aging is often the consequence of a neurodegenerative disease, such as dementia, that is a progressive illness characterized by changes in different brain regions, such as inferior and middle temporal structures and parietal structures in case of Alzheimer’s disease (Pettigrew et al., 2017), and consequentially—changes in cognitive functioning (ICD-11 for Mortality and Morbidity Statistics, n.d.). While the brain and cognitive changes in normal aging still allow the individual to remain fully functional in their daily lives, pathological aging involves significant impairment and even disability and with time require professional assistance.

Even though pharmacological approaches to the treatment of neurodegenerative diseases, such as Alzheimer’s Disease, have been extensively investigated, no definite treatment has been found. Nevertheless, more and more studies indicate the role of cognitive reserve as a means of prolonging the functionally healthy years of older adults (Livingston et al., 2020).

### 1.1 The reserve theory

In the late 1980’s Robert Katzman and colleagues published a study investigating clinical, pathological and neurochemical changes in dementia, discussing results of *postmortem* examination on 137 older adults from a nursing facility. The results indicated that part of the subjects, who had a diagnosis of Alzheimer’s Disease (AD) had similar brain characteristics when compared to the control group without AD, thus suggesting that these patients might have better brain reserve (Katzman et al., 1988). Later studies were conducted in early 1990’s, finding lower education, lower occupational attainment and lower intelligence to be risk factors for cognitive abnormalities in HIV-1 positive patients (Satz et al., 1993). These findings were later complemented in 1999 by Yaakov Stern, showing that higher educational and occupational attainments were associated with more rapid memory decline when compared with patients who already presented with low memory scores, thus proposing a theoretical model explaining the effect of cognitive reserve (Stern et al., 1999; Stern, 2009). These results were later supported by other studies, providing an approximation of the predicted cognitively healthy years after clinical onset of the (Marioni et al., 2012). This effect was proposed to be associated with two concepts – brain and cognitive reserve These concepts are considered to be the two sides of the same coin, where cognitive reserve refers to the learnt capacity of using the brain to overcome a cognitive or intellectual challenge or cope better with a pathology, whereas brain reserve is considered the ‘hardware’ and is measured by the neurological capacity, e.g., brain volume or synaptic count (Stern, 2002; Stern et al., 2020). The interaction between both concepts is still largely unknown, with studies showing a relationship between sociobehavioral proxies of cognitive reserve, such as education and occupation and different brain structures; however, while brain reserve often has been measured using estimated intracranial brain volume as a proxy, it is a very rough approximate and further studies are needed (van Loenhoud et al., 2018).

More recently the new concept of brain maintenance was defined. Brain maintenance refers to the relative lack of changes in the brain structural and neurochemical properties or lack of neuropathology over time and often can serve as a protective factor against cognitive decline (Alvares Pereira et al., 2022; Stern et al., 2023). The brain maintenance could be associated with unmodifiable factors, such as genes and sex, as well as early life influences, sociobehavioral factors, e.g., education, and environmental factors. Nevertheless, it should be stressed that the concept can not be completely separated from the concepts of cognitive reserve and brain reserve and still should be considered to be complementary to each other (Nyberg et al., 2021; Stern et al., 2023).

### 1.2 Lifestyle associated sociobehavioral proxies of cognitive reserve

One of the first and still most often used approaches in measuring cognitive reserve, is the sociodemographic approach that is based on the assumption that cognitive reserve is built through life-style experiences, such as educational and occupational achievements, leisure activities, language abilities etc. While the use of proxies as measures of cognitive reserve has been criticized, mostly regarding to the limitations of measurement psychometric properties and overall differences in measures (Kartschmit et al., 2019), they still provide an overall understanding of cognitive reserve. In this study, education, occupation and leisure activities were used as proxies of cognitive reserve.

Education is one of the first and also the most commonly used proxy of cognitive reserve and several studies have found the relationship between lower levels of education and higher risk of developing dementia or Alzheimer’s disease—a claim supported by several cross-sectional studies (Sharp and Gatz, 2011; Takasugi et al., 2021), nevertheless, longitudinal studies indicate that, although education could influence cognitive performance before the onset of normal or clinical decline, it does not predict changes in cognitive performance or brain volume (Wilson et al., 2019; Nyberg et al., 2021).

Occupation complexity is another significant factor that has been present since early studies. Studies have shown that involvement in more complex occupational activities is associated with better retainment of existing cognitive capacity (Mondini et al., 2022), even if the individual is already considered to be a risk group (Boots et al., 2015). While overall there are definite benefits of being employed, such as developing new skills, social engagement, daily routine etc. (Vance et al., 2016), some studies have also pointed toward specific occupational demands targeting specific cognitive domains (Spreng et al., 2011).

Leisure activities could potentially be the most challenging proxy of cognitive reserve, as there is still no consensus regarding the activities that should be included. Some studies have indicated that social, cultural and cognitive activities could decrease the risk of developing dementia increasing cognitive performance (Wajman et al., 2018), still most of the studies do not specify type, regularity and when during life-time these activities have to be carried out.

### 1.3 Lifestyle activities and cortical volume

There are two main approaches to mapping the function to the specific region of the brain. Traditionally, the segregation approach has been used, according to which the cerebral cortex can be subdivided into distinct regions that can be distinguished based on functional and structural properties. Another approach to mapping brain and behavior is the integration approach that postulates that no brain region is sufficient to execute a function by itself, thus there should be a dynamic interplay between the regions (Genon et al., 2018). While both approaches indicate an opposite relationship (structure vs. interaction), it should be noted that they do not exclude each other but are, in fact symbiotic, namely the regions might be locally segregated; however, globally integrated (Deco et al., 2015; Wang et al., 2021). Recently, a study investigating the effect of cognitive reserve on structural and functional magnetic resonance measures were conducted in healthy young adults, indicating a modulating effect on various brain motor and cognitive networks (Conti et al., 2021). Similar studies have been conducted mapping cognitive reserve in patients with Alzheimer’s disease, showing a potential relationship between the whole brain and temporoparietal atrophies (van Loenhoud et al., 2017).

Previous studies have indicated relationships between life-style measures and cortical volumetry in both – healthy and demented individuals. Earlier studies have indicated a relationship between years spent obtaining formal education and cortical volume in right hemisphere superior temporal gyrus, left hemisphere insular cortex, bilateral anterior cingulate gyrus (Arenaza-Urquijo et al., 2013), transverse temporal cortex, the insula and the isthmus (Liu et al., 2012). Several studies have also considered the relationship between occupational activities and cortical measures, such as thickness, showing that more complex physical occupations could be associated with higher cortical thickness in the primary motor and somatosensory cortex (Lenhart et al., 2021), nevertheless, the amount of studies considering occupational activities and cortical volume, is sparse.

**The aim** of this study is to investigate the potential relationship between lifestyle socio-behavioral proxies of cognitive reserve and cortical regions in adults with no subjective cognitive decline. We hypothesized that higher indices on the Cognitive Reserve Index questionnaire subscales will be associated with larger cortical volume in different regions.

## 2 Methodology

### 2.1 Participants

In total 70 older adults with no subjective cognitive decline participated in the study, 23.9% male, 65–85 years of age (Mage = 72.19, SD = 5.02). Four participants were excluded from the study due to not meeting all the inclusion / exclusion criteria, two were removed due to the inability to have MRI, and six showed significant outliers in one or several variables. All outliers were identified using box plots and based on the SD. As a result, 58 participants, aged 65 to 85 years, were included in the data analysis (*M* = 71.83, SD = 5.02, 20.7% male).

#### 2.1.1 Inclusion/exclusion criteria

In data acquisition and analysis, only participants aged 65+ and daily Latvian speakers were included. Furthermore, we included participants who did not reported currently experiencing neurological, cardiovascular, pulmonary and/or respiratory diseases that require inhalators, ongoing oncological disease, rheumatologic diseases that require pain medication, ongoing mental disease (e.g., depression or dementia), as well as did not indicate any other factors, such as metallic implants. To ensure that participants comply with the criteria, all prospective participants were interviewed prior to inviting them to take part in the study, determining their overall health status (are there any of the aforementioned diseases), age and language. Compliance with health criteria (yes/no) were stored in a password-protected document.

### 2.2 Measures

To assess the lifestyle associated socio-behavioral proxies of **cognitive reserve**, the Cognitive Reserve Index questionnaire (Nucci et al., 2012) translated and adapted in Latvian language was used. CRIq is a semi-structured interview aiming to estimate the cognitive reserve obtained through life and consists of three main domains: formal and informal education (CRI Education), working activity, defined by the level of complexity and responsibility (CRI Occupation), and leisure activities (including weekly, monthly, annual, and fixed activities) (CRI Leisure Activity), thus allowing one to evaluate cognitive reserve using a combined sociodemographic approach instead of a single proxy. A total index combining all three proxies can be calculated as well (CRI Total). Higher indices indicate a higher cognitive reserve in the respective domain.

**General cognitive abilities** were assessed using the Montreal Cognitive Assessment (Nasreddine et al., 2005) scale that is a brief screening instrument for assessing and identifying mild cognitive impairment or dementia. The scale includes six main cognitive domains—attention and focus, executive functions, memory, verbal abilities, visuo-spatial abilities and orientation in space and time. Only the overall score was used in data analysis.

**Figure 1:**
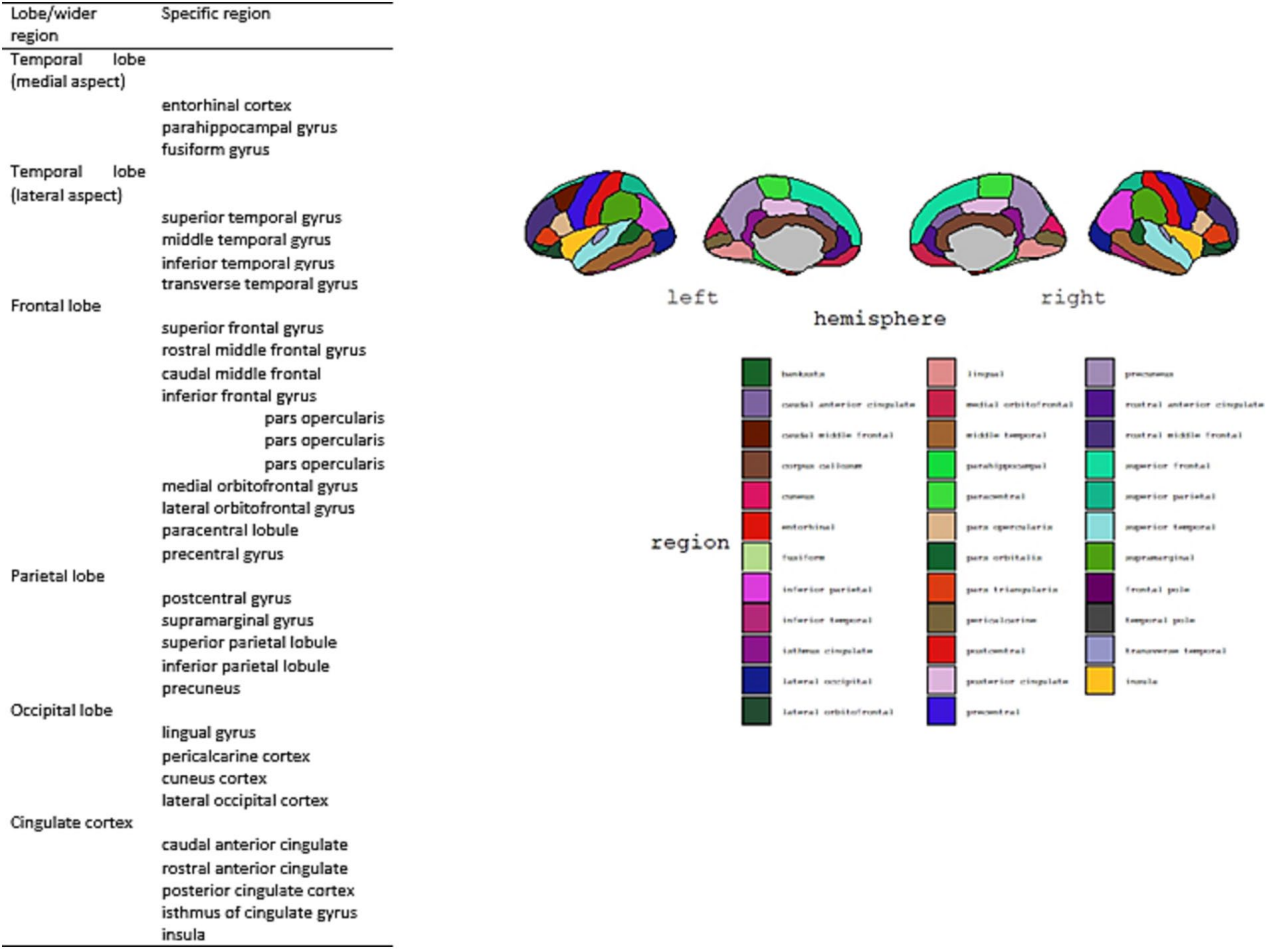
Desikan-Killiany-Tourville (DKT) Atlas structures (Alexander et al., 2019) and visual representation of DKT regions [developed with ggseg package (Mowinckel and Vidal-Piñeiro, 2020)].

**Cortical volumetry measures** were obtained using a Siemens 1.5 Tesla Avanto MRI scanner (Siemens, Germany) and analysis was later conducted using Freesurfer 7.2. version and applying Desikan-Killiany-Tourville (DKT) Atlas (Alexander et al., 2019, see Figure 1). High-resolution anatomical images were acquired using a three-dimensional T1-weighted magnetization prepared rapid acquisition gradient echo (MPRAGE) sequence [TR = 1,160 ms; TE = 4.44 ms; inversion recovery time (TI) = 600 ms; field of view (FOV), 230 × 230 mm2; matrix size, 256 × 256; flip angle θ = 15 degrees; voxel dimensions, 0.9 × 0.9 × 0.9 mm3; acquisition time, 5 min].

### 2.3 Procedure

Data were acquired from each participant individually. Furthermore, sociodemographic data and cognitive assessment were conducted, as well as the data obtained on lifestyle socio-behavioral proxies of cognitive reserve. MRI acquisition was performed in collaboration with Pauls Stradiņš Clinical University Hospital. Before MRI, all participants confirmed that they had no metallic implants or other problems that could pose a risk for MRI acquisition.

To ensure the compliance with the principles of research ethics, approval from Riga Stradiņš University Ethics Committee was obtained. All participants signed an informed consent form, where they were introduced to the objectives, risks, and benefits of the study, as well as explained that all data will be confidential and only coded versions of the data will be available.

### 2.4 Statistical analysis

To determine the initial relationship between proxies of cognitive reserve and cortical regions, Spearman rank correlation coefficient was used, due to the small sample size and non-normal data distribution. To further investigate the relationship between the variables after controlling for estimated intracranial volume and age, hierarchical linear regression analysis was used. In addition, to identify the effect of cognitive reserve, moderation analysis using continuous moderator was conducted with R 4.3.0. version.

## 3 Results

### 3.1 Descriptive statistics

Median, mean, sd, min and max values were obtained for cognitive reserve associated life-style proxies (see Table 1).

**Table 1:** Descriptive statistics of lifestyle associated proxies of cognitive reserve.

Variable	Median	Mean	SD	Min	Max
CRI education	122.5	121	11.58	98	151
CRI occupation	117	120.74	20.97	88	176
CRI leisure	131.5	131.47	15.58	90	172
CRI total	132	132.33	16.08	98	175

### 3.2 Association between lifestyle associated proxies of cognitive reserve and cortical regions

**Figure 2:**
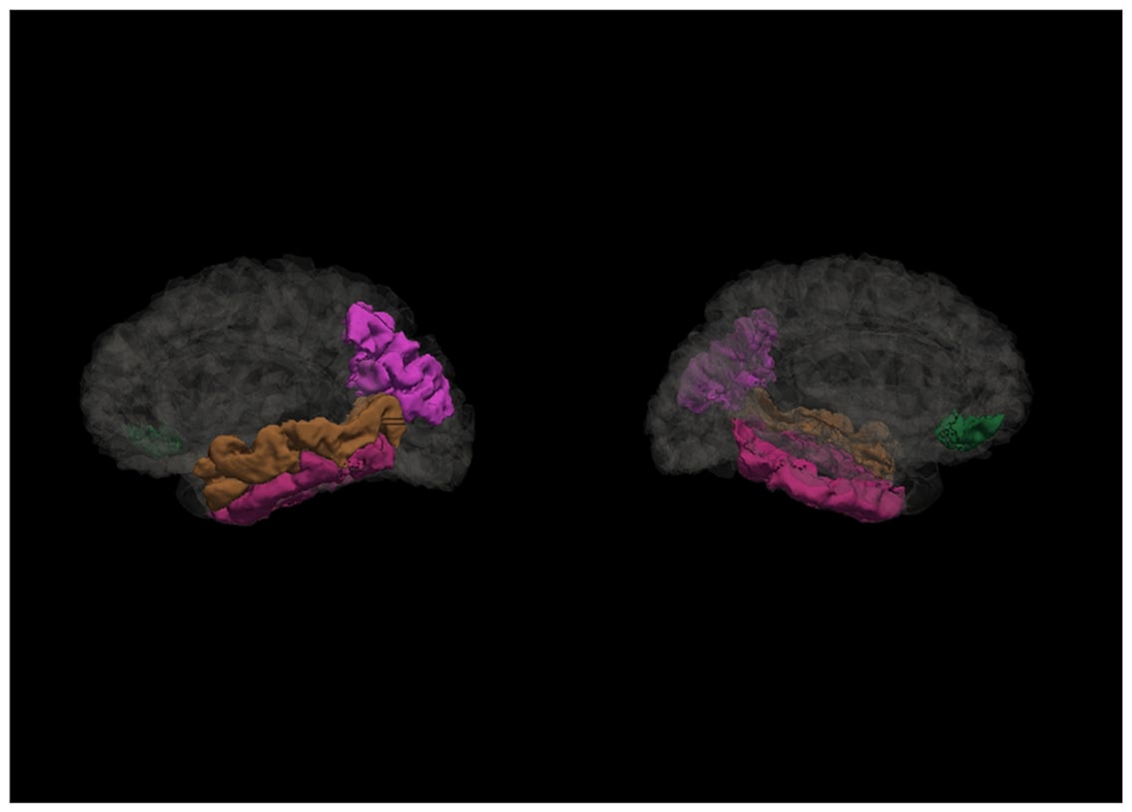
Regions associated with cognitive reserve and its proxies. This figure illustrates the cortical regions associated with occupational cognitive reserve and total cognitive reserve score after controlling for eTIV and age (sagittal view of left and right hemispheres, accordingly).

The Spearman rank correlation analysis showed a statistically significant relationship between CRI-Education, CRI-Occupation, and CRI-Total scores and regions in the temporal, frontal, parietal, occipital and cingulate gyrus (see Supplementary Tables S1–S6). Nevertheless, the associations changed significantly when controlling for age and estimated intracranial volume (eTIV) (see the associated regions in Figure 2). CRI-Education no longer indicated a relationship with any of the cortical regions; CRI-Occupation, however, was significantly related to several temporal and parietal regions. Regarding the temporal regions, CRI-Occupation was associated with left middle temporal gyrus [*R*^2^ = 0.396, *ΔR*^2^ = 0.063, *F*(1, 54) = 5.627, *p* = 0.021], explaining 6.3% of the variation in the regional volume and inferior temporal gyrus, explaining 9.6% of the regional volume [*R*^2^ = 0.292, *ΔR*^2^ = 0.096, *F*(1, 54) = 7.336, *p* = 0.009]. CRI-Occupation also was significantly associated with the right inferior temporal gyrus, explaining 8.5% of the regional volume [*R*^2^ = 0.406, *ΔR*^2^ = 0.085, *F*(1, 54) = 7.765, *p* = 0.007] and with the regional volume in left inferior parietal lobule [*R*^2^ = 0.483, *ΔR*^2^ = 0.138, *F*(1, 54) = 14.404, *p* < 0.001] explaining 13.8% of the variation (see Table 2).

**Table 2:** Relationship between CRI-Occupation and cortical regions after controlling for age and eTIV (linear regression analysis).

Dependent variable	*R*^2^	Δ*R*^2^	*B*	SE B	β	*F*	*p*
lh middle temporal	0.396	0.063	18.252	7.694	0.252	5.627	0.021
lh inferior temporal	0.292	0.096	21.532	7.950	0.312	7.336	0.009
rh inferior temporal	0.406	0.085	18.514	6.644	0.294	7.765	0.007
lh inferior parietal	0.483	0.138	24.920	6.566	0.373	14.404	0.001

CRI-Total was associated with temporal, frontal, and parietal regions, specifically the left middle temporal gyrus and inferior parietal lobule and right pars orbitalis (see Table 3). Similarly as with CRI-Occupation, also the total cognitive reserve score was associated with left middle temporal gyrus and inferior parietal lobule, explaining 6.3 and 12% of the variation accordingly; however, CRI-total was also statistically significantly related to right pars orbitalis, explaining 8.4% of the volume variation volume [*R*^2^ = 0.127, *ΔR*^2^ = 0.084, *F*(1, 54) = 5.223, *p* = 0.026].

**Table 3:** Relationship between CRI-Total and cortical regions after controlling for age and eTIV (linear regression analysis).

Dependent variable	*R*^2^	Δ*R*^2^	*B*	SE B	β	*F*	*p*
lh middle temporal	0.396	0.063	23.752	9.988	0.252	5.656	0.021
rh pars orbitalis	0.127	0.084	4.556	1.994	0.291	5.223	0.026
lh inferior parietal	0.465	0.120	30.173	8.672	0.347	12.107	0.001

To better understand the role of lifestyle socio-behavioral proxies in healthy aging, moderation analysis was conducted using volumetry indicators for cortical regions as independent variables, MoCA scores as dependent variable and socio-behavioral proxies of CR as moderator variables, nevertheless, no moderation effect was found (*p* > 0.05).

## 4 Discussion

This study aimed to investigate the potential relationship between lifestyle socio-behavioral proxies of cognitive reserve and cortical regions in adults with no subjective cognitive decline. We hypothesized that higher indices of the lifestyle associated socio-behavioral proxies, as measured using a composite index, will be associated with larger cortical volume, especially, in regions associated with pathology related cognitive decline (e.g., inferior temporal regions and medial temporal regions). The results indicate a potential interaction between lifestyle measures as cognitive reserve proxies and cortical regions. Participants involved in occupational activities that include higher professional competence and higher work responsibility showed larger cortical volume in the left middle temporal gyrus, the bilateral inferior temporal gyrus, and the left inferior parietal lobule, while combined proxies (the CRI Total combined score that includes educational achievement, occupation and leisure activities) showed a positive relationship with the volume of left middle temporal gyrus and inferior parietal lobule, and pars orbitalis in the right hemisphere.

Previous studies using neuroimaging methods and different cognitive reserve methodology also indicate the relationship between CR and cortical and subcortical structures. A study by van Loenhoud et al. (2017) indicated that cognitive reserve as measured by standardized individual differences between predicted and observed gray matter and validated by education was associated with greater atrophy in the whole brain and temporoparietal atrophy in participants with Alzheimer’s disease, thus confirming that these regions could be more sensitive to the effects of CR. Another study investigating the possible association between CR and brain regions showed that a higher global CR (composite score consisting of leisure activities, verbal IQ and education) did not find any association with temporal and parietal regions, but rather with the middle frontal gyrus (orbital part), the supplementary motor area, and the cerebellum of the left hemisphere (Conti et al., 2021). Although these findings do not directly agree with our results, they complement our findings and it should be noted that their composite proxy did not include occupation.

Surprisingly, in our data after controlling for age and estimated intracranial volume (eTIV), education did not show a relationship with cortical regions. Historically, education was considered the main proxy for cognitive reserve (see, e.g., Satz et al., 1993), possibly due to the association between lower levels of education and neurodegenerative disease (Norton et al., 2014), however, recent studies aim to define it as a factor in setting a threshold, rather than having longitudinal impact (Cadar et al., 2017; Wilson et al., 2019; Nyberg et al., 2021). Meanwhile, more and more attention has been paid to other potential factors that facilitate cognitive reserve, with occupational activity being one of them. It should be stressed that in many high-level professions, higher education is essential; thus, it could be hypothesized that the educational element is still present, but strengthened by occupational activities that are usually engaged during life time as oppose to education. It should also be noted that in general our participants showed higher educational achievements in general that could have impact on the overall results.

Higher occupational achievements as well as higher total cognitive reserve index was associated with a larger volume of regions commonly associated with linguistic capabilities, namely, **middle temporal gyrus** and **pars orbitalis**. A recent study conducted by Boyle et al. (2021) indicated that verbal intelligence could be a more precise proxy measure of cognitive reserve, in comparison to sociodemographic proxies. Verbal intelligence has been associated with temporal regions, in particular, in the left hemisphere (Heyer et al., 2021), thus this might indicate verbal IQ as supplementary for sociodemographic composite scores when measuring cognitive reserve. It should be noted that while the correlation analysis of our study also indicated a relationship between occupation, total CRI, and left pars orbitalis (traditionally associated with language production), after controlling for age and eTIV the regression analysis showed no significant relationship. Nevertheless, results of the hierarchal regression analysis indicated an association between total CRI and right **pars orbitalis**, a region whose functional role is still debated. Some indications of the relationship between right pars orbitalis and verbal learning and fluency has been found in patients in early stages of psychotic illness, but not in controls (Kenney et al., 2017). While there is a gap in studies considering pars orbitalis in the non-dominant hemisphere, studies including the right inferior frontal gyrus – area within which lays pars orbitalis – has been shown to be vulnerable to different types of dementia and thus might benefit from lifestyle activities or interventions (Schroeter et al., 2012).

Occupation was also associated with a larger bilateral **inferior temporal gyrus**. The inferior temporal gyrus has been highly associated with visual processing, and previous connectome studies have also indicated a potential relationship between CR and the inferior temporal gyrus, showing greater functional connectivity between CR measures and the occipital regions, centrality of the inferior temporal gyrus and greater global efficiency (Marques et al., 2016). It should be noted that cortical thinning in middle and temporal gyrus has been identified in adults with subjective cognitive decline (Rivas-Fernández et al., 2023). Connectome studies have also identified the **inferior parietal lobe** as one of the functional rich club hubs in the brain (Oldham and Fornito, 2019). It could be hypothesized that higher occupational complexity could contribute to strengthening at least one of the hubs, thus improving the efficiency of complex cognitive processes; however, additional studies are needed to test this hypothesis.

Previous studies indicate the vulnerability of temporal and parietal regions to Alzheimer’s disease - areas including lateral temporal structures – inferior temporal gyrus, middle temporal gyrus and parietal structures, such as the superior and inferior parietal gyrus (Pettigrew et al., 2016). The results of our study complement the data of Pettigrew et al. (2017), showing that cognitive reserve might be related to the same regions, thus supporting a potential protective effect against AD. Further studies should be considered in clinical samples, including not only AD patients, but also Parkinson’s disease and dementia in Parkinson’s disease, as controversial results have been found in studies exploring this relationship. While some studies have indicated a positive effects of cognitive reserve on lowering the risk of longitudinal progression to mild cognitive impairment in Parkinson’s disease (Gu and Xu, 2022) and having a modulatory effect on functional connectivity in basal ganglia and executive-attentional fronto-parietal networks (Di Tella et al., 2023), others find no effect in the long-term (Hindle et al., 2014).

Considering that cognitive reserve is still a rather theoretical concept, it is difficult to measure it directly, therefore, typically socio-behavioral proxies are used in combination with other measures, such as, cognitive performance (Stern et al., 2020). We conducted moderation analysis, using socio-behavioral proxies as moderators, nevertheless, no effect was found on the relationship between specific cortical regions and cognitive functioning. This could be related to the overall higher scores in cognitive performance and in CRIq indices, nevertheless, repeated analysis in a larger sample with participants showing significant structural and cognitive decline could be beneficial to better understand the effect of cognitive reserve. It could be argued that the current results are more representative of the concept of brain maintenance, however, cognitive reserve and brain maintenance are complementary concepts (Stern et al., 2023) and in this study our focus lies in predicting the changes, rather than the constancy.

A note of caution is due here, since the volumetric data were obtained with a 1.5 Tesla machine, thus reducing the quality of images. This is also significant when considering the delicate nature of structures, e.g., one region might have different functions depending on more detailed segmentation. In addition, this study would benefit from functional connectivity measures (e.g., using fMRI) that could be the object of further studies. In addition, while we recognize that the results of the inferential tests could emerge as significant by chance (i.e., Type I error or “false positive”), no Bonferroni correction was applied to this study. Nevertheless, this was a conscious choice, as in smaller samples this increases a risk of Type II error, namely, “false negative” (e.g., see Armstrong, 2014). The authors also recognize the limitations proposed by the sample size, nevertheless it should be stressed that overall, the results of our study comply with the findings from other individual studies.

Although cognitive reserve was measured using a well-developed tool combined from a variety of sociodemographic factors, it should still be stressed that this questionnaire has a high risk of memory bias, especially with respect to leisure activities. Furthermore, in our sample, all indices showed a tendency toward higher scores that could affect the results of the study. Taking into account these limitations, further studies would benefit from a more heterogeneous and a larger sample that would include participants with lower education and less complex occupational activities. To lessen the potential impact of memory bias, additional approaches to measuring CR could be integrated that would complement the sociodemographic approach.

## Data availability statement

The raw data supporting the conclusions of this article will be made available by the authors, without undue reservation.

## Ethics statement

The studies involving humans were approved by Riga Stradiņš University Ethics Committee. The studies were conducted in accordance with the local legislation and institutional requirements. The participants provided their written informed consent to participate in this study.

## Author contributions

KS: Conceptualization, Data curation, Formal Analysis, Methodology, Writing – original draft. NZ: Investigation, Software, Visualization, Writing – review & editing. SM: Conceptualization, Supervision, Writing – review & editing. AS: Conceptualization, Funding acquisition, Project administration, Supervision, Writing – review & editing.
