# Supplementary material for: Relationship between lifestyle proxies of cognitive reserve and cortical regions in older adults
Source: Front Psychol. 2024 Jan 5;14:1308434. doi: 10.3389/fpsyg.2023.1308434 (PMC10797127; doi:10.3389/fpsyg.2023.1308434)
Supplement: Supplementary file 1 [file Table_1.docx]

Supplementary Material

**Table A.1**

*Spearman rank correlation between cognitive reserve and temporal lobe (medial aspect) regions*

| *Variable* | 1. | 2. | 3. | 4. | 5. | 6. | 7. | 8. | 9. | 10. |
| --- | --- | --- | --- | --- | --- | --- | --- | --- | --- | --- |
| 1. CRI Education | -- |  |  |  |  |  |  |  |  |  |
| 1. CRI Occupation | .377** | -- |  |  |  |  |  |  |  |  |
| 1. CRI Leisure | .281* | .258 | -- |  |  |  |  |  |  |  |
| 1. CRI Total | .670** | .817** | .627** | -- |  |  |  |  |  |  |
| 1. lh entorhinal | -.032 | .127 | -.163 | -.032 | -- |  |  |  |  |  |
| 1. rh entorhinal | .203 | **.336*** | .011 | .230 | .672** | -- |  |  |  |  |
| 1. lh parahippocampal | .079 | .071 | -.055 | .038 | .542** | .410** | -- |  |  |  |
| 1. rh parahippocampal | .094 | . 178 | -.007 | .105 | .474** | .534** | .568** | -- |  |  |
| 1. lh fusiform | -.031 | .114 | -.178 | -.033 | .328** | .541** | .265* | .338** | -- |  |
| 1. rh fusiform | -.039 | .208 | -.105 | .043 | .343** | .536** | .333* | .278* | .587** | -- |

*Note.* lh – left hemisphere, rh – right hemisphere, * - *p* < .05, ** - *p* < .01

**Table A.2**

*Spearman rank correlation between cognitive reserve and temporal lobe (lateral aspect) regions*

| *Variable* | 1. | 2. | 3. | 4. | 5. | 6. | 7. | 8. | 9. | 10. | 11. | 12. |
| --- | --- | --- | --- | --- | --- | --- | --- | --- | --- | --- | --- | --- |
| 1. CRI Education | -- |  |  |  |  |  |  |  |  |  |  |  |
| 1. CRI Occupation | .377** | -- |  |  |  |  |  |  |  |  |  |  |
| 1. CRI Leisure | .281* | .258 | -- |  |  |  |  |  |  |  |  |  |
| 1. CRI Total | .670** | .817** | .627** | -- |  |  |  |  |  |  |  |  |
| 1. lh superior | .066 | **.345**** | -.162 | .131 | -- |  |  |  |  |  |  |  |
| 1. rh superior | .115 | **.316*** | -.039 | .187 | .826** | -- |  |  |  |  |  |  |
| 1. lh middle | .198 | **.384**** | .033 | **.259*** | .529** | .543** | -- |  |  |  |  |  |
| 1. rh middle | .197 | .229 | -.026 | .147 | .544** | .580** | .684** | -- |  |  |  |  |
| 1. lh inferior | .207 | **.417**** | .012 | .258 | .429** | .544** | .578** | .523** | -- |  |  |  |
| 1. rh inferior | .171 | **.450**** | -.016 | .255 | .525** | .565** | .646** | .452** | .607** | -- |  |  |
| 1. lh transverse | -.224 | -.050 | -.078 | -.073 | .277* | .266* | .138 | -.049 | -.035 | .237 | -- |  |
| 1. rh transverse | -.011 | .285* | .057 | .193 | .610** | .623** | .334* | .396** | .298* | .345** | .409** | -- |

*Note.* lh – left hemisphere, rh – right hemisphere, * - *p* < .05, ** - *p* < .01

**Table A.3**

*Spearman rank correlation between cognitive reserve and frontal lobe regions*

| *Variable* | 1. | 2. | 3. | 4. | 5. | 6. | 7. | 8. | 9. | 10. | 11. | 12. | 13. | 14. | 15. | 16. | 17. | 18. | 19. | 20. | 21. | 22. | 23. | 24. |
| --- | --- | --- | --- | --- | --- | --- | --- | --- | --- | --- | --- | --- | --- | --- | --- | --- | --- | --- | --- | --- | --- | --- | --- | --- |
| 1. CRI Education | -- |  |  |  |  |  |  |  |  |  |  |  |  |  |  |  |  |  |  |  |  |  |  |  |
| 1. CRI Occupation | .377** | -- |  |  |  |  |  |  |  |  |  |  |  |  |  |  |  |  |  |  |  |  |  |  |
| 1. CRI Leisure | .281* | .258 | -- |  |  |  |  |  |  |  |  |  |  |  |  |  |  |  |  |  |  |  |  |  |
| 1. CRI Total | .670** | .817** | .627** | -- |  |  |  |  |  |  |  |  |  |  |  |  |  |  |  |  |  |  |  |  |
| 1. lh superior frontal | .066 | .162 | -.204 | .016 | -- |  |  |  |  |  |  |  |  |  |  |  |  |  |  |  |  |  |  |  |
| 1. rh superior frontal | .100 | .257 | -.122 | .139 | .752 | -- |  |  |  |  |  |  |  |  |  |  |  |  |  |  |  |  |  |  |
| 1. lh rostral middle | -.048 | **.306*** | -.101 | .084 | .714 | .622 | -- |  |  |  |  |  |  |  |  |  |  |  |  |  |  |  |  |  |
| 1. rh rostral middle | .024 | .164 | -.207 | .020 | .568 | .584 | .689 | -- |  |  |  |  |  |  |  |  |  |  |  |  |  |  |  |  |
| 1. lh caudal middle | .041 | -.060 | -.019 | -.046 | .246 | .243 | .076 | .148 | -- |  |  |  |  |  |  |  |  |  |  |  |  |  |  |  |
| 1. rh caudal middle | .221 | .142 | -.044 | .158 | .310 | .292 | .260 | .257 | .405 | -- |  |  |  |  |  |  |  |  |  |  |  |  |  |  |
| 1. lh pars opercularis | -.051 | .200 | -.062 | .045 | .494 | .446 | .634 | .434 | .243 | .327 | -- |  |  |  |  |  |  |  |  |  |  |  |  |  |
| 1. rh pars opercularis | .056 | .111 | -.171 | .009 | .316 | .390 | .319 | .486 | .110 | .276 | .503 | -- |  |  |  |  |  |  |  |  |  |  |  |  |
| 1. lh pars triangularis | -.130 | .031 | -.126 | -.066 | .491 | .388 | .558 | .341 | -.160 | .212 | .751 | .336 | -- |  |  |  |  |  |  |  |  |  |  |  |
| 1. rh pars triangularis | .034 | .058 | -.098 | .008 | .383 | .316 | .333 | .380 | -.025 | .002 | .485 | .581 | .485 | -- |  |  |  |  |  |  |  |  |  |  |
| 1. lh pars orbitalis | .063 | **.296*** | -.032 | .188 | .544 | .445 | .491 | .321 | -.030 | .149 | .351 | .235 | .490 | .453 | -- |  |  |  |  |  |  |  |  |  |
| 1. rh pars orbitalis | .111 | **.323*** | **.339**** | **.411**** | .188 | .268 | .143 | .241 | -.038 | -.004 | .023 | .106 | .090 | .096 | .452 | -- |  |  |  |  |  |  |  |  |
| 1. lh medial orbitofrontal | .140 | .106 | -.028 | .045 | .657 | .537 | .605 | .404 | .157 | .166 | .484 | .349 | .402 | .354 | .479 | .093 | -- |  |  |  |  |  |  |  |
| 1. rh medial orbitofrontal | .242 | **.285*** | -.005 | .243 | .485 | .458 | .438 | .395 | .060 | .311 | .229 | .174 | .292 | .206 | .446 | .466 | .441 | -- |  |  |  |  |  |  |
| 1. lh lateral orbitofrontal | .216 | **.327*** | -.036 | .224 | .678 | .592 | .564 | .353 | .097 | .337 | .380 | .303 | .381 | .353 | .631 | .318 | .587 | .619 | -- |  |  |  |  |  |
| 1. rh lateral orbitofrontal | .186 | **.274*** | .101 | .241 | .644 | .494 | .607 | .360 | .164 | .291 | .456 | .130 | .437 | .232 | .528 | .356 | .581 | .664 | .679 | -- |  |  |  |  |
| 1. lh paracentral lobule | -.141 | .160 | -.139 | -.030 | .610 | .425 | .550 | .335 | .272 | .363 | .334 | .174 | .297 | .155 | .454 | .136 | .586 | .532 | .582 | .473 | -- |  |  |  |
| 1. rh paracentral lobule | **-.293*** | -.025 | -.171 | -.195 | .465 | .367 | .388 | .251 | .310 | .125 | .227 | .100 | .107 | .117 | .368 | .132 | .529 | .394 | .414 | .401 | .571 | -- |  |  |
| 1. lh precentral gyrus | .045 | -.093 | .047 | .001 | .096 | .200 | -.121 | .077 | .377 | .253 | -.159 | .005 | -.186 | -.013 | .142 | .337 | .176 | .305 | .150 | .031 | .427 | .337 | -- |  |
| 1. rh precentral gyrus | .024 | -.098 | -.068 | -.096 | .450 | .341 | .246 | .065 | .507 | .473 | .224 | .014 | .177 | .056 | .256 | .030 | .438 | .391 | .373 | .367 | .600 | .535 | .559 | -- |

*Note.* lh – left hemisphere, rh – right hemisphere, * - *p* < .05, ** - *p* < .01

**Table A.4**

*Spearman rank correlation between cognitive reserve and parietal lobe regions*

| *Variable* | 1. | 2. | 3. | 4. | 5. | 6. | 7. | 8. | 9. | 10. | 11. | 12. | 13. | 14. |
| --- | --- | --- | --- | --- | --- | --- | --- | --- | --- | --- | --- | --- | --- | --- |
| 1. CRI Education | -- |  |  |  |  |  |  |  |  |  |  |  |  |  |
| 1. CRI Occupation | .377** | -- |  |  |  |  |  |  |  |  |  |  |  |  |
| 1. CRI Leisure | .281* | .258 | -- |  |  |  |  |  |  |  |  |  |  |  |
| 1. CRI Total | .670** | .817** | .627** | -- |  |  |  |  |  |  |  |  |  |  |
| 1. lh postcentral | .096 | .095 | -.003 | .101 | -- |  |  |  |  |  |  |  |  |  |
| 1. rh postcentral | .041 | -.008 | .013 | .022 | .689 | -- |  |  |  |  |  |  |  |  |
| 1. lh supramarginal | .076 | -.007 | .159 | .066 | .732 | .498 | -- |  |  |  |  |  |  |  |
| 1. rh supramarginal | .117 | .250 | .085 | .179 | .734 | .683 | .613 | -- |  |  |  |  |  |  |
| 1. lh superior parietal | .105 | **.262*** | .120 | .229 | .390 | .250 | .442 | .291 | -- |  |  |  |  |  |
| 1. rh superior parietal | .090 | .114 | .068 | .066 | .380 | .413 | .384 | .621 | .430 | -- |  |  |  |  |
| 1. lh inferior parietal | .233 | **.471**** | .172 | **.387**** | .274 | .329 | .174 | .397 | .354 | .221 | -- |  |  |  |
| 1. rh inferior parietal | .051 | .195 | -.054 | .071 | .274 | .343 | .150 | .318 | .074 | .340 | .502 | -- |  |  |
| 1. lh precuneus | .055 | .105 | .003 | .071 | .489 | .432 | .454 | .305 | .653 | .198 | .234 | .137 | -- |  |
| 1. rh precuneus | .096 | .033 | -.057 | -.010 | .600 | .615 | .506 | .511 | .451 | .482 | .234 | .444 | .660 | -- |

*Note.* lh – left hemisphere, rh – right hemisphere, * - *p* < .05, ** - *p* < .01

**Table A.5**

*Spearman rank correlation between cognitive reserve and occipital lobe regions*

| *Variable* | 1. | 2. | 3. | 4. | 5. | 6. | 7. | 8. | 9. | 10. | 11. | 12. |
| --- | --- | --- | --- | --- | --- | --- | --- | --- | --- | --- | --- | --- |
| 1. CRI Education | -- |  |  |  |  |  |  |  |  |  |  |  |
| 1. CRI Occupation | .377** | -- |  |  |  |  |  |  |  |  |  |  |
| 1. CRI Leisure | .281* | .258 | -- |  |  |  |  |  |  |  |  |  |
| 1. CRI Total | .670** | .817** | .627** | -- |  |  |  |  |  |  |  |  |
| 1. lh lingual | .066 | .013 | -.176 | -.015 | -- |  |  |  |  |  |  |  |
| 1. rh lingual | .163 | .094 | .050 | .137 | .523 | -- |  |  |  |  |  |  |
| 1. lh pericalcarine | .014 | -.151 | -.071 | -.045 | .507 | .565 | -- |  |  |  |  |  |
| 1. rh pericalcarine | .218 | .202 | .054 | **.285*** | .344 | .681 | .687 | -- |  |  |  |  |
| 1. lh cuneus | -.043 | -.162 | -.211 | -.157 | .368 | .393 | .678 | .502 | -- |  |  |  |
| 1. rh cuneus | -.008 | -.037 | -.219 | -.046 | .491 | .550 | .658 | .668 | .627 | -- |  |  |
| 1. lh lateral occipital | .050 | .137 | -.114 | .025 | .255 | .240 | .139 | .169 | .202 | .312 | -- |  |
| 1. rh lateral occipital | -.118 | .028 | -.257 | -.116 | .353 | .106 | .166 | .121 | .391 | .376 | .690 | -- |

*Note.* lh – left hemisphere, rh – right hemisphere, * - *p* < .05, ** - *p* < .01

**Table A.6**

*Spearman rank correlation between cognitive reserve and cingulate regions*

| *Variable* | 1. | 2. | 3. | 4. | 5. | 6. | 7. | 8. | 9. | 10. | 11. | 12. | 13. | 14. |
| --- | --- | --- | --- | --- | --- | --- | --- | --- | --- | --- | --- | --- | --- | --- |
| 1. CRI Education | -- |  |  |  |  |  |  |  |  |  |  |  |  |  |
| 1. CRI Occupation | .377** | -- |  |  |  |  |  |  |  |  |  |  |  |  |
| 1. CRI Leisure | .281* | .258 | -- |  |  |  |  |  |  |  |  |  |  |  |
| 1. CRI Total | .670** | .817** | .627** | -- |  |  |  |  |  |  |  |  |  |  |
| 1. lh caudal anterior | .114 | **.266*** | .034 | .211 | -- |  |  |  |  |  |  |  |  |  |
| 1. rh caudal anterior | -.036 | .218 | -.099 | .081 | .621 | -- |  |  |  |  |  |  |  |  |
| 1. lh rostral anterior | .203 | **.320*** | .084 | .244 | .628 | .256 | -- |  |  |  |  |  |  |  |
| 1. rh rostral anterior | **.272*** | **.301*** | .032 | **.274*** | .495 | .623 | .388 | -- |  |  |  |  |  |  |
| 1. lh posterior cingulate | **.266*** | .155 | .074 | .229 | .675 | .434 | .503 | .477 | -- |  |  |  |  |  |
| 1. rh posterior cingulate | -.012 | .141 | .002 | .096 | .621 | .563 | .381 | .473 | .701 | -- |  |  |  |  |
| 1. lh isthmus | .251 | .062 | .180 | .237 | .448 | .148 | .387 | .351 | .565 | .469 | -- |  |  |  |
| 1. rh isthmus | .195 | .116 | -.145 | .115 | .483 | .263 | .493 | .336 | .494 | .450 | .607 | -- |  |  |
| 1. lh insula | .239 | **.261*** | .137 | **.294*** | .481 | .254 | .595 | .512 | .520 | .476 | .425 | .415 | -- |  |
| 1. rh insula | .238 | .247 | .188 | **.309*** | .437 | .157 | .572 | .438 | .486 | .476 | .441 | .477 | .906 | -- |

*Note.* lh – left hemisphere, rh – right hemisphere, * - *p* < .05, ** - *p* < .01
